# Exploring molecular and cellular signaling pathways: Unraveling the pathogenesis of tendinopathy

**DOI:** 10.1016/j.jot.2025.02.003

**Published:** 2025-03-20

**Authors:** Zihan Xu, Wenjing Hou, Tao Zhang, Rui Chen, Thomas Skutella

**Affiliations:** a Department of Neuroanatomy, Group for Regeneration and Reprogramming, Institute for Anatomy and Cell Biology, Medical Faculty, Heidelberg University, 69120, Heidelberg, Germany; b Department of Reproductive Medicine, The Affiliated Hospital of Qingdao University, Qingdao, Shandong, 266000, China

**Keywords:** Extracellular matrix, Pathogenesis, Tendon, Tendinopathy, Tendon stem/progenitor cell

## Abstract

Despite the long healing duration of tendon injuries, the outcomes of repairs are frequently suboptimal, resulting in persistent pain and reduced functionality. Current clinical approaches to tendinopathy are primarily symptomatic, encompassing nonsteroidal anti-inflammatory drugs (NSAIDs), corticosteroid injections, physical therapies, surgical interventions, loading programs, and pain management. Yet, these treatments have protracted timelines and their efficacy remains uncertain. This uncertainty stems largely from an incomplete understanding of tendinopathy's pathogenesis. Unraveling the mechanisms behind tendinopathy is essential for devising novel therapeutic strategies. In this context, this review systematic reviewed more recent cellular and molecular literature in tendinopathy, in order to summarize the up-to-date advancements including the structure and composition of healthy tendons, the pathophysiological changes in tendinopathy, the molecular pathways implicated in various forms of the condition, and current effective treatment methods. This review not only aims to offer insights but also to inspire further investigation into the mechanisms and clinical management of tendinopathy.

**The translational potential of this article:**

A deficient understanding of the molecular mechanisms hampers the advancement of therapeutic strategies and drug development. Consequently, an in-depth examination of these molecular mechanisms is essential for comprehending the etiology of tendinopathy and for devising effective clinical management strategies.

## Graphical abstract

**Figure:**
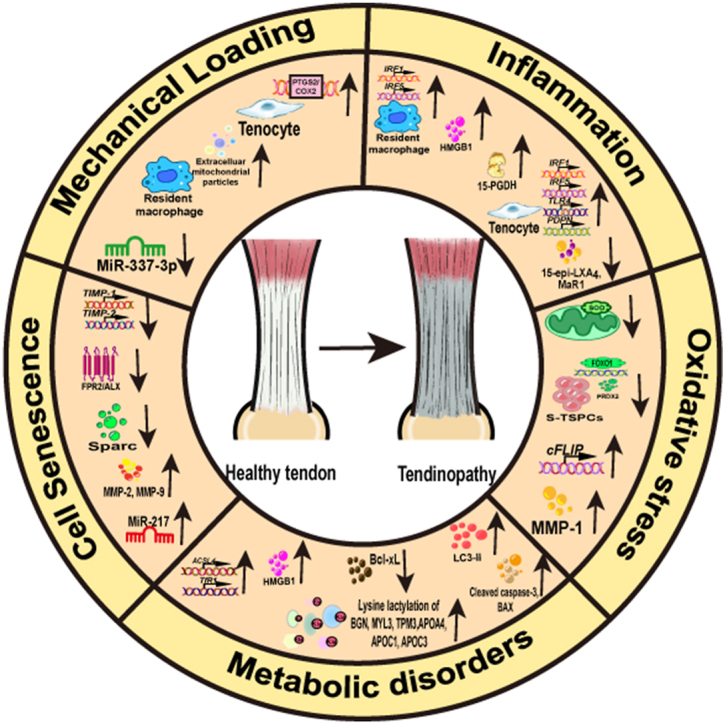

## 1 Background

Tendon disorders encompass a spectrum of conditions, from traumatic injuries to chronic degenerative diseases such as tendinopathies. These conditions can induce alterations in the histological and molecular characteristics of the tendons. Clinically, such alterations present as a constellation of symptoms, including pain, swelling, and a decline in functional capacity [1]. The development of tendinopathy can be broadly attributed to intrinsic and extrinsic factors. Intrinsic factors encompass a spectrum of conditions such as systemic diseases, familial predispositions, advancing age, impairments in neuromuscular control, irregular joint mobility, muscular weakness, tendinosis, and structural alterations within the tendon. Extrinsic factors are environmental and activity-related, including overuse injuries, improper intensity of physical activity, inadequate recovery periods, and poor workplace ergonomics [2]. Historically, inconsistencies in clinical nomenclature have hindered the recognition of the pathological basis of tendon conditions. In 1998, Maffulli et al. took a significant step towards standardization by advocating using the term 'tendinopathy' as a general clinical descriptor. This terminology encompasses several specific classifications, including tendinitis, tenosynovitis, and tendinosis, providing a more unified approach to diagnosing and discussing these conditions [3]. Over the past few decades, more individuals have been engaging in sports and exercise for health benefits or recreational purposes. Consequently, tendon injuries has escalated [[4], [5], [6]] as common orthopedic injuries, tendinopathies and traumatic tendon injuries account for over 30% of all musculoskeletal consultations [7]. Furthermore, there appears to be a current gap in the reported prevalence of tendinopathy. This is because patients who are asymptomatic during examination may go unnoticed. Research indicates that when diagnostic methods incorporate radiological imaging, such as ultrasound, the identified incidence rate of tendinopathy is significantly higher than when such imaging is not utilized [8,9]. Additionally, a higher tolerance for illness and disability in the elderly may be another reason for this discrepancy [10]. The prevalence and incidence of tendinopathy vary between genders, with some forms of the condition exhibiting a higher occurrence in one sex than the other [11,12]. A higher number of cases in males or females may not reflect which gender is more prone to developing tendinopathies, these gender-based discrepancies may depend on the different sports preferences and different physical loading on the tendon, or possibly on the interaction of these factors with gender-specific pathophysiological differences [[12], [13], [14]]. Despite increasing participation in various sports over recent decades, data to subdivide specific pathology between genders is still insufficient. There continues to be a general lack of literature examining how females are affected by tendinopathy [15]. Patients with tendinopathies are generally older than the general practice population. Population aged between 45 and 64 are the largest affected [11]. However, tendinopathies can also occur at an early age [9,16]. In addition, due to the occupational demands, specific populations who perform repetitive activities more often, more intensely, and for a longer time face higher incidences of tendinopathy. For example, the incidence of the midportion Achilles tendinopathy in the general population is 1.85 per 1000 general practitioners, this is lower than the incidence of 2.98 found in the military population, and then the annual incidence of 7 % and 9 % in top-level runners [17]. Multiple management strategies, including surgical repairs, pain management, various physical therapies, loading programs, etc., have been recommended for treatments in patients with tendinopathy [18,19]. Although these approaches act through divergent mechanisms, their shared goal is to reduce symptoms, in particular, to reduce pain, promote tendon healing, and improve patient function, the outcomes are not always satisfactory and are correlated with inferior repair capacity of the tendon and poor understanding of pathological changes [20]. Severe tendinopathy can hamper mobility and refined movement, making people retire early with decreased well-being, resulting in an economic burden on patients and society. Furthermore, lacking understanding of molecular mechanisms impedes the development of therapeutic approaches and agents. Therefore, reviewing in-depth studies of these molecular mechanisms is necessary in understanding the pathogenesis of tendinopathy and in identifying the methods for clinical management.

## 2 Molecular composition of healthy tendons

Understanding the intricate molecular composition of a healthy tendon is crucial for our collective knowledge. It provides a solid foundation for comprehending the deviations in tendinopathy, empowering us to devise effective therapeutic strategies.

Tendons, as robust fibrous connective tissues, serve as essential links between muscles and bones, playing a pivotal role in transmitting tensile forces generated by muscle contractions, facilitating a wide range of bodily movements [21]. Their structural organization is a marvel of three-dimensional hierarchy. The primary subunit of tendons, the bundles, are irregularly shaped, with diameters ranging from 150 to 500 μm, they are composed of an intricate network of collagen fibers, each ranges from 1 to 20 μm in diameter [22]. These fascicles are bound together by the interfascicular matrix (IFM), as endotenon. This connective tissue embeds blood vessels and nerves integral to the complete tendon unit [23]. The epitenon, a connective tissue sheath, interfaces with the endotenon and envelopes the tendon's periphery [22]. The collagen fibers are further subdivided into fibrils, which are 10–500 nm in diameter, made up of microfibrils, or pentafibrils, comprising five intertwined tropocollagen molecules [1]. Enveloping the fascicle bundle is the paratenon, a loose connective tissue layer that aids in the gliding movement of the tendon, with the epitenon and paratenon collectively termed peritenon [24].

Tendons are composed of both cellular components and an extracellular matrix (ECM), with variations in composition and structure, particularly pronounced at the myotendinous junction (MTJ) and the osteotendinous junction (OTJ) [25]. The MTJ is a highly specialized region that bridges the tension from muscle fibers to extracellular connective tissue proteins, specifically collagen fibrils. Conversely, the OTJ is where the tendon integrates with the bone, characterized by four distinct zones: tendon, fibrocartilage, mineralized fibrocartilage, and bone [26]. The predominant cellular element of tendon tissue is the mature, elongated tenocytes, which derive from immature, spindle-shaped tenoblasts with large, oval nuclei, aligning uniformly along the collagen fibrils' long axis and constituting 90–95 % of the cellular content. These tenocytes are chiefly responsible for producing ECM components, such as collagen, fibronectin, and proteoglycans, which are vital for maintaining tendon homeostasis and repairing injured tendons [27]. The fibroblasts within the IFM area are interfascicular tenocytes, while those within the fascicle bundles are termed intrafascicular tenocytes [22]. The remaining cellular constituents include rounded 'fibrochondrocyte' cells, synovial cells of the tendon sheath, smooth muscle cells, endothelial cells of arterioles, and sparse nerve fibers and endings [28]. The fibrochondrocyte cells are typically found in the fibrocartilaginous regions, often in columnar arrangements at the OTJ, while synovial cells are situated between the endotenon and epitenon. Nerve fibers are usually near blood vessels, traversing through the endotenon and epitenon [29].

Tendons also contain a small population of cells with stem cell characteristics, such as self-renewal, clonogenicity, and differentiation potential, which are crucial for tendon development, homeostasis, and healing. These are tendon stem/progenitor cells (TSPCs) [24]. TSPCs from various niches, including the tendon proper, peritenon, and perivascular regions, exhibit distinct multipotency in vitro, identified by different molecular markers [30,31]. The proper-derived TSPCs show increased expression of tenomodulin (Tnmd) and scleraxis (Scx), indicating their tendon origin. In contrast, peritenon-derived TSPCs exhibit higher levels of vascular and pericyte markers relative to those from the tendon proper [32]. In response to injury, perivascular TSPCs migrate into the interstitial space and contribute to ECM deposition [31]. The distinction between TSPCs and tenocytes is not well-defined, with a lack of consensus on specific markers, leading to their identification and study challenges. Li et al. proposed of using Scx, Tnmd, and Col I for tenocyte identification and CD90 and CD44 in conjunction with these markers for TSPC identification [33]. A clearer understanding and classification of TSPCs are necessary for advancing tendon research and therapy [34]. In addition, a small population of resident macrophages is found within the tendon. These cells are typically quiescent and self-renew under physiological conditions [35,36]. However, upon tendon injury, they become activated, secreting inflammatory factors such as tumor necrosis factor-alpha (TNF-α), interleukin-1β(IL-1β), and IL-6, and actively participating in the repair process. They are considered key mediators in the formation of scar tissue [37,38]. Specifically, macrophages differentiate into M1 and M2 phenotypes, with M1 macrophages secreting pro-inflammatory mediators and M2 macrophages contributing to the resolution of inflammation and tissue remodeling. The transition from M1 to M2 phenotypes is crucial for the progression from inflammation to tissue repair and scar formation [39,40]. Additionally, macrophage-derived miRNA-containing exosomes have been implicated in peritendinous fibrosis after tendon injury through the miR-21-5p/Smad7 pathway, suggesting a complex role of macrophages in the tendon healing process and scar tissue formation [41].

The tendon ECM is a complex structure composed of three main classes of biomolecules: structural proteins (collagen and elastin), specialized proteins (e.g., fibrillin and fibronectin), and proteoglycans [1]. Collagens form the bulk of the mature tendon ECM, constituting 65–80 % of its dry weight, and are primarily responsible for the tendon's tensile resistance [42]. Collagen I (Col I) accounts for nearly 95 % of the total collagen content [34,43]. Collagen II (Col II) is localized to the tendon fibrocartilage at the OTJ, while collagen XXII (Col XXII) is specifically localized at MTJ and produced by muscle cells and serves as a cell adhesion ligand for fibroblasts [44], and collagen III (Col III) is found in the endotenon and epitenon [45,46]. Moreover, during development, collagen XI plays a role in regulating the formation of collagen fibrils and is broadly expressed in tendons [47]. Other collagen types, including Col IV, V, VI, IX, X, XII, and XIV, are also in smaller quantities [1]. Elastin, constituting 1–2% of the tendon's dry weight, is the main component of elastic fibers, providing the tendon with extensibility [48]. Fibrillins, large extracellular glycoproteins, form the core of microfibrils and offer a scaffold for elastin deposition [49]. Fibronectin, an adhesive glycoprotein, plays a crucial role in fibrillogenesis and matrix assembly, binding to cell surface receptors and ECM components. Fibronectin, an adhesive glycoprotein, plays a vital role in fibrillogenesis and matrix assembly, binding to cell surface receptors and ECM components [50]. Proteoglycans, hydrophilic protein-polysaccharide complexes, facilitate the diffusion of water-soluble molecules and cell migration [51]. The interplay between the cellular components and the ECM is bidirectional, both elements essential for maintaining the tendon's healthy development and biomechanical properties.

## 3 Macroscopic and microscopic observations of tendinopathy

According to its anatomical sites within the tendon, tendinopathy shows different manifestations. The MTJ is rarely affected by tendinopathy, the OTJ is often affected at or near the insertion in the fibrocartilaginous such as the supraspinatus tendon and patellar tendon, while the mid-portion of the tendon is affected and commonly observed in the Achilles tendon [52]. A critical vital feature of these sites is their heightened stress levels compared to the different areas of the tendon regions.

Macroscopic examination of insertional tendinopathy, such as in the supraspinatus tendon, often reveals hyaline degeneration, mucoid degeneration, and the presence of calcium deposits [53]. Tendons are characterized by a distinct crimped pattern of collagen fibers that act as natural shock absorbers. However, tendinopathy leads to the disarray and separation of collagen bundles [54]. Microscopically, insertional tendinopathy is typified by hypocellularity, vacuolation, nuclear pyknosis, a more uniform matrix, and reduced eosinophilia, with no evident inflammation [55,56]. Occasionally, fibrocartilaginous metaplasia is also observed. Despite the absence of clinical inflammation, modern molecular techniques have unveiled the involvement of immune cells and inflammatory processes in tendinopathy across various models. Tendon biopsies from recent studies have shown increased transcription of inflammatory cytokines, alarmin production, and immune cell recruitment and activation, indicating the role of inflammation in the progression of rotator cuff tendinopathy [[57], [58], [59], [60]].

In the case of mid-portion tendinopathy, such as in the Achilles tendon, histopathological studies have uncovered collagen fiber degeneration and disarray. Symptomatic Achilles tendons may also exhibit haphazardly increased blood vessels [61]. The degeneration in these tendons is often mucoid or lipoid, with mucoid degeneration causing the region to soften and change from a glistening white color to grey or brown. Light microscopy shows thinner collagen fibers, large mucoid patches, vacuoles and the Alcian blue-stained ground substance. Lipoid degeneration is abnormal lipid accumulation within the tendon tissue [26].

In 2008, Cook JL et al. proposed a pathology model for load-induced tendinopathy, outlining a three-stage continuum: reactive tendinopathy, tendon disrepair (failed healing), and degenerative tendinopathy [62]. During the reactive phase, the tendon's cross-sectional area increases due to cell proliferation and matrix metaplasia, an adaptive response to reduce stress. During this phase, and the tendon cells take on a chondroid-like shape with enhanced protein production capabilities [63]. The primary proteins upregulated are proteoglycans, which can rapidly alter the matrix due to increased bound water and contribute to the fibrosis process [64]. The tendon may revert to its normal state if proper rest is taken [62]. Failure in the initial healing process leads to tendon disrepair, characterized by greater, more significant, matrix breakdown, thickening of the tendon, discontinuity of collagen fascicles, and potential increases in vascularity and neuronal ingrowth [65]. Some reversibility is still possible at this stage with appropriate load management and exercise to promote matrix recovery [66]. The degenerative stage is marked by extensive cellular and matrix degeneration, with cells undergoing apoptosis and the matrix becoming disordered and populated by vessels, mucoid ground substance, and reparative collagen (Col III) [67]. The tendon shows signs of damage, with irregular crimping, fiber loosening, and increased waviness, and has minimal healing capacity [68]. Unfortunately, there is little capacity for tendon healing at this stage.

In conclusion, pathological changes in tendinopathy include a range of conditions that affect the structure and function of tendons. Specifically, fibrosis and ectopic ossification are two common pathological changes observed in tendinopathy. Fibrosis is characterized by the replacement of normal tendon tissue with fibrous connective tissue, leading to a loss of the tendon's normal structure and function. The pathological changes associated with fibrosis include a reduction in the number of fibroblasts and rounding of the remaining cells, an increase in the content of proteoglycans within the ECM, and a disruption of the normal collagen fiber arrangement [69]. Ectopic ossification in tendinopathy is characterized by chondrogenesis and ossification of the tendon. The pathological changes display similarities towards fibrocartilaginous metaplasia and calcium hydroxyapatite deposit, as shown by the acquired chondroid features of the tenocytes with a rounded appearance and a prominent nucleus [70]. Occasionally, neovascularization occurs, which is the formation of new blood vessels in the affected area. So, the pathological changes in tendinopathy are complex, highly variable, and contingent upon the lesion location and developmental phase, painting tendinopathy as a degenerative disease of the tendon with a spectrum of manifestations and complexities that require nuanced understanding and treatment strategies.

## 4 Molecular pathogenesis of tendinopathy

The traditional view posits that tendinopathy arises primarily from mechanical overload and repetitive strain [69]. Yet, a more contemporary understanding acknowledges the multifactorial and intricate nature of tendinopathy's pathogenesis, encompassing a spectrum of intrinsic and extrinsic factors. The interplay between these factors, either in isolation or in concert, fuels the development of tendinopathy. Recent strides in molecular biology and genomics have cast light on the molecular underpinnings of this condition, deepening our insight into its etiology and unveiling potential therapeutic targets.

### 4.1 Mechanical loading

**Fig. 1:**
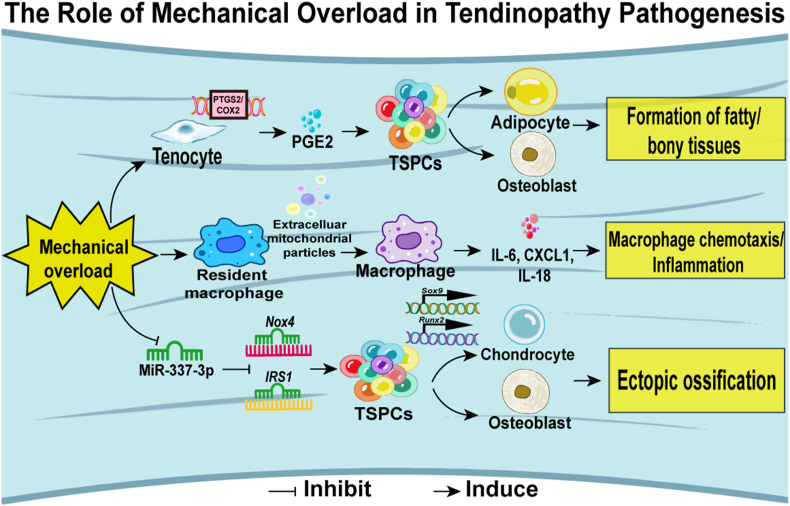
The pathogenesis of tendinopathy regarding mechanical overload. Current research indicates that mechanical overload can result in the formation of adipose tissue, calcification, inflammation, and ectopic ossification within tendon by these mechanisms.

Mechanical forces play a pivotal role in tenocyte morphogenesis and are essential to the appropriate development and maintenance of tendons. Subramanian and colleagues have elucidated that the activation of TGF-β signaling in response to these forces is critical for the growth and branching of tenocyte projections [71]. In instances where muscle contraction is absent, as in paralyzed embryos, there is a notable loss of phosphorylated SMAD3 expression, a key intracellular signal transducer. This deficit can be mitigated by reinitiating muscle contraction, underscoring the necessity of mechanical stimulation for tendon health. (see. Fig. 1)

However, when mechanical loading becomes excessive or repetitive, it can trigger the onset of tendinopathy. Abraham and their team, through the creation of a murine model mimicking rotator cuff tendinopathy, have identified a suppression in the expression of wound healing-related genes and a significant upregulation of prostaglandin-endoperoxide synthase 2 (PTGS2), a gene associated with the degeneration and improper differentiation of tendon progenitor cells [72,73]. In an innovative murine model of subacromial impingement, which examines supraspinatus tendinopathy, a downregulation of interleukin-1α (IL-1α) was observed in the impingement group compared to the control group. This study also highlighted previously unreported genes that contribute to tendinopathies, such as secreted phosphoprotein 1 (Spp1), Tnn (Tenascin N), and growth arrest DNA damage-inducible gene 45 (Gadd45g), offering new insights into the molecular players involved in this condition [74].These findings underscore how aberrant mechanical signaling can lead to pathological changes within the tendon. In addition, mechanical loading has been shown to induce the release of extracellular particles from tendon cells, which can incite macrophage chemotaxis and amplify the production of proinflammatory cytokines, suggesting a mechanistic link between mechanical stress and the inflammatory response in tendinopathy development [75]. The role of mechanical forces extends to the late stages of tendinopathy, where heterotopic ossification can arise, impacting the tendon's mechanical properties and homeostasis. Intriguingly, miR-337-3p, a mechanosensitive microRNA, has been found to have reduced expression under uniaxial cyclical mechanical loading in tendon-derived stem cells (TDSCs), negatively regulating their chondro-osteogenic differentiation. This regulatory mechanism is linked to the proinflammatory cytokine interleukin-1β, which acts as an upstream regulator of miR-337-3p, thereby connecting mechanical stress to the miRNA's expression and its role in ectopic ossification in tendinopathy [76]. The influence of mechanical load on tendon health is bidirectional, while excessive load can degrade tendons, controlled loading is also a common therapeutic approach. Tendons subjected to prolonged underloading exhibit increased osteogenic gene expression and heterotopic ossification alongside diminished biomechanical strength. This phenomenon is associated with increased β-catenin accumulation and altered phosphorylation patterns, offering insights into the molecular responses to mechanical underloading [77]. Conversely, isometric loading following a central core tendon injury has been shown to reduce cell protection, enabling tendon cells to experience tensile stress that can enhance healing, as indicated by elevated markers such as Scx and Col I. In contrast, dynamic loading protocols that mimic physiological conditions can induce a response indicative of compression in the tendon matrix, as seen in the upregulation of Col II [78]. Furthermore, mechanical stretching has been observed under high-glucose conditions to initiate calcium influx, activating the CaMKK2/AMPK signaling pathway. This activation leads to the reorganization of the FAK-cytoskeleton and promotes the expression of EGR1, a protein integral to maintain tendon cell properties and their balance. These findings illuminate the role of mechanobiology in maintaining tendon cell stability, particularly in the context of diabetic tendinopathy [79].

### 4.2 Inflammation

**Fig. 2:**
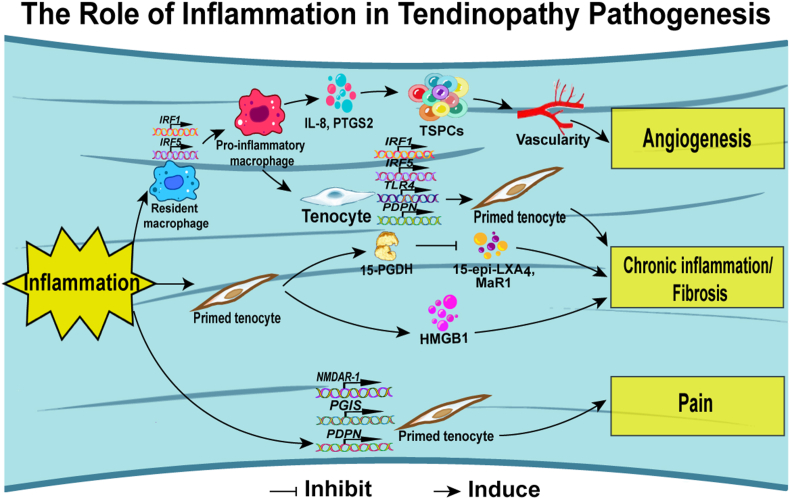
Mechanisms of tendinopathy concerning to inflammation. Current research suggests that inflammation can lead to tendon angiogenesis, chronic inflammation, fibrosis, and pain through these mechanisms.

Inflammation is a critical mediator in the pathogenesis of tendinopathy. Inflammation characterizes both tendinopathy and tendon rupture. A study examined the expression of target molecules from these pathways in healthy and diseased Achilles tendon tissues and found a complex inflammatory signature in patient samples, with expression of molecules from interferon, such as nuclear factor kappa-B (NF-κB), Signal transducer and activator of transcription-6 (STAT-6), and glucocorticoid receptor (GCR) pathways, indicating chronic inflammation and tissue repair [60]. Notably, tendinopathic Achilles tendons exhibited heightened expression of interferon-related genes such as interferon regulatory factor 1 (IRF1), interferon regulatory factor 5 (IRF5), and chemokine ligand 10 (CXCL10), echoing patterns seen in tendinopathic rotator cuff tissues. In the case of ruptured Achilles tendons, an intense expression of NF-κB-related genes PTGS2 and IL-8 was observed, correlated with increased vascularity and angiogenesis—a phenomenon not mirrored in rotator cuff tears. Moreover, Dakin et al. unearthed a novel facet of tendinopathy, revealing that diseased tendon cells harbor elevated levels of the enzyme 15-prostaglandin dehydrogenase (15-PGDH) alongside increased breakdown products of aspirin-triggered lipoxin A4 (15-epi-LXA4) and maresin 1 (MaR1). The strategic use of drugs to inhibit this enzyme modulated the conversion of these bioactive lipid mediators, thereby helping to regulate inflammatory markers interleukin-6 (IL-6), podoplanin (PDPN), and signal transducer and activator of transcription-1 (STAT-1). This discovery suggests a potential imbalance in the production of lipid mediators, typically involved in the resolution of inflammation and may contribute to chronic inflammatory states within tendinopathic tissues [80]. (see.Fig. 2)

High-mobility group protein B1 (HMGB1), an alarmin released by cells under duress, operates as a danger-associated molecular pattern (DAMP), inciting a flurry of pro-inflammatory cytokines, cell proliferation, and matrix responses and may significantly contribute to musculoskeletal disorders [81]. Intriguingly, HMGB1 is detectable in the early stages of tendinopathy, with tenocytes identified as a principal source of this protein. It appears to be intricately involved in tendon cell inflammation and matrix regulation through toll-like receptor 4 (TLR4) signaling [82]. Exogenous HMGB1 has also been implicated in tendinopathy, promoting the translocation of HMGB1 from the nucleus to the cytoplasm, igniting NF-κB and mitogen-activated protein kinase (MAPK) signaling pathways, and fostering hyper-cellularity and immune cell infiltration in rat Achilles tendon tissues [83]. S100A8 and S100A9, additional alarmins secreted by immune cells such as monocytes and neutrophils, are found in increased concentrations within tendinopathic tissues. Experiments with human tenocytes have demonstrated that the introduction of S100A8 and S100A9 augments the release of inflammatory proteins, suggesting their participation in a positive feedback loop that amplifies leukocyte recruitment and the secretion of pro-inflammatory cytokines, thus perpetuating the inflammatory response in the early stages of tendinopathy [84]. The impact of prostaglandins on inflammation and pain in tendinopathy has also been a subject of intense research. Diseased tendon tissues have shown heightened prostacyclin receptor (IP) expression and enzymes involved in prostaglandin biosynthesis, including cyclooxygenase-1 (COX-1), cyclooxygenase-2 (COX-2), prostacyclin synthase (PGIS), and microsomal prostaglandin E synthase-1 (mPGES-1). PGIS was found to co-localize with cells expressing PDPN and the nociceptive neuromodulator N-methyl-D-aspartate receptor 1 (NMDAR-1), indicating that prostaglandin E2 (PGE2) may perpetuate inflammation and pain. At the same time, prostacyclin could offer a protective effect in human tendon disease [85].

Epigenetics also influences the onset, progression, and modulation of inflammation. Trella and colleagues identified five genes (Leprel2, Foxf1, MMP25, Igwp6, and Peg12) that showed a strong correlation in a murine model of Achilles tendinopathy [86]. In a genome-wide association study, Kim and colleagues pinpointed two single nucleotide polymorphisms (SNPs) within the cytochrome c oxidase assembly factor 1 (COA1) gene that may be associated with the development of patellar tendinopathy [87]. The discovery of these genes and SNPs may be instrumental in revealing the underlying mechanisms of tendinopathy.

### 4.3 Oxidative stress

**Fig. 3:**
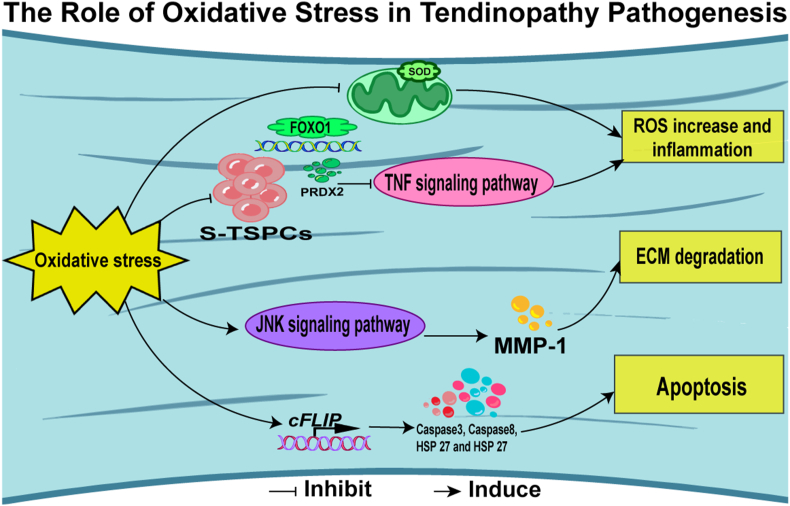
Mechanisms of tendinopathy stemming from oxidative stress. Current research indicates that oxidative stress can contribute to inflammation, degradation of the extracellular matrix, and apoptosis via these mechanisms.

Oxidative stress is a significant contributor to tendinopathy, particularly in intense physical exertion. This phenomenon arises when the body's innate immunity that defenses against harmful oxygen molecules, known as reactive oxygen species (ROS), are overwhelmed. Excess ROS can catalyze detrimental oxidative modifications to cellular components, leading to cellular dysfunction and tissue damage. The observed upregulation of the antioxidant enzyme peroxiredoxin 5 (PRDX5), which plays a protective role against oxidative stress, suggests a significant presence of oxidative stress in human tendinopathy [88]. Proteomic analyses of specimens from tendinopathy patients have revealed upregulation of proteins such as S100A11, PLIN4, HYOU1, and CLIC1, intimately associated with oxidative stress and chronic inflammation [89].(see Fig. 3)

Utilizing an innovative device that simulates both physiological and mechanically overloaded conditions, researchers observed more pronounced increases in the expression of peroxisome proliferator-activated receptor gamma (Pparg) and Sox9 in the 8% strain group compared to the 4 % strain group. Interestingly, while the gene expression of Col I remained consistent between groups, the expression of Col III and the Col III/Col I ratio was elevated in the 8% strain group. The application of anti-oxidative medications, such as epigallocatechin gallate and piracetam, has been shown to reduce the expression of genes indicating non-tenocyte lineage differentiation (Pparg, Sox9, and Runx2) in cellular models of tendinopathy [90]. In a multi-modal analysis combining single-cell RNA and ATAC sequencing with the tendinopathy landscape, J. Guo et al. identified a specific cell subpopulation with low Peroxiredoxin 2 (PRDX2) expression, which exhibited heightened inflammation, diminished proliferation, and migration capabilities. Moreover, the tumor necrosis factor (TNF) signaling pathway was significantly activated in these cells. Forkhead box protein O1 (FOXO1), an upstream regulator of PRDX2 transcription, forms a potential regulatory mechanism called the FOXO1-PRDX2-TNF axis, for treating tendinopathy. TNF silencing significantly reversed the increase in intracellular ROS and mitigated the inhibition of the proliferation and aging of TDSCs [91]. To elucidate the nexus between rotator cuff degeneration and oxidative stress, a study in antioxidant enzyme superoxide dismutase 1 (Sod1)-deficient mice (Sod1^−/−^) demonstrated that the loss of Sod1 leads to the downregulation of collagen and a decrease in the mechanical properties of the supraspinatus enthesis. This finding implies that intracellular oxidative stress contributes to the degeneration of rotator cuff entheses [92]. In a similar vein, an evaluation of mitochondrial dysfunction in supraspinatus tendinopathy confirmed that mitochondrial Sod, which neutralizes the highly reactive superoxide radical, is diminished during the development of tendinopathy [93]. Furthermore, matrix metallopeptidase-1 (MMP-1) activity, a key driver of tendon matrix degradation, can be upregulated by phosphorylated c-Jun amino-terminal kinase (JNK) induced by oxidative stress, leading to tendon matrix degradation [94,95]. Oxidative stress-induced apoptosis also plays a crucial role in tendon degeneration. Heat shock proteins (HSPs) often upregulated in response to oxidative and other forms of stress, are elevated in tendinopathy tissue. The reduction of the apoptotic gene cFLIP, the increase in caspases 3 and 8 and HSPs 27 and 70, suggests that heat shock proteins are integral to the cascade of stress-activated programmed cell death and degeneration in tendinopathy [96].

### 4.4 Tendon cell senescence

**Fig. 4:**
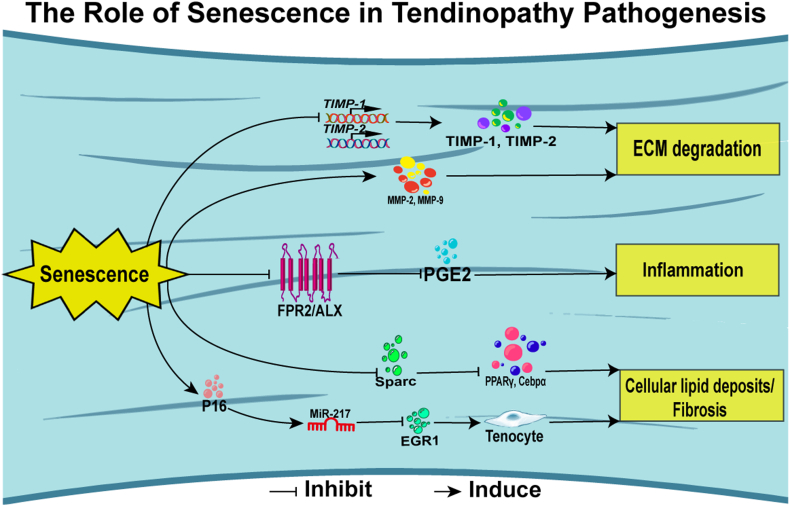
Pathogenesis of tendinopathy associated cell senescence. Current research suggests that senescence can lead to inflammation, degradation of the extracellular matrix, and cellular lipid deposition and fibrosis through these mechanisms.

Senescent cells, characterized by their secretion of matrix-degrading enzymes and pro-inflammatory cytokines—collectively termed senescence-associated secretory phenotypes (SASPs)—have been implicated in the pathogenesis of tendinopathy. These SASPs contribute to the deterioration of the ECM and the propagation of inflammation, which are critical factors in developing tendinopathic conditions.(see Fig. 4)

The expression of matrix metalloproteinases (MMPs), enzymes crucial for matrix remodeling, is influenced by mechanical strain and is linked to the loss of ECM integrity observed in chronic tendon diseases. Dudhia et al. discovered that the levels of both pro- and active forms of MMP-2 and MMP-9 were elevated in aged tissues compared to their younger counterparts [97]. This trend was corroborated by the findings of Tung-Yang Yu and colleagues, who noted a significant age-related increase in the enzymatic activities of MMP-2 and -9. In addition, a comparative analysis between young and aging tenocytes revealed a marked decrease in the mRNA levels of tissue inhibitors of metalloproteinases 1 and 2 (TIMP-1 and TIMP-2) in the aging group [98]. A study delving into the roles of prostaglandins and inflammation-resolving mediators in equine tendon injury highlighted the impact of age on the resolution of tendon inflammation. The researchers analyzed the levels of PGE2, PGF2α, lipoxin A4 (LXA4), and its receptor formyl peptide receptor type 2/lipoxin A4 receptor (FPR2/ALX) in normal average, sub-acute, and chronically injured tendons. They found that aging individuals have a diminished capacity to resolve tendon inflammation via the FPR2/ALX receptor, suggesting a potential mechanism for the progression of chronic tendinopathy [99]. Furthermore, the capacity for tenogenic differentiation in TSPCs declines with age, which may contribute to the development of tendinopathy. Senescence in TSPCs is associated with a reduction in their tenogenic differentiation potential. The senescence marker p16 has been shown to inhibit the tenogenic differentiation of TSPCs through miRNA-217 and its target early growth response factor 1 (EGR1) [100]. An investigation into the role of aging in the erroneous differentiation of TSPCs post-injury revealed that aging inhibits adipogenesis of TSPCs by downregulating peroxisome proliferator-activated receptor γ (PPARγ) signaling, indicating alternative pathways may lead to adipocyte accumulation in tendinopathy [101]. Gehwolf et al. highlighted the age-related decrease in the expression of secreted protein acidic and rich in cysteine (Sparc). This loss results in defects in tendon collagen fibrillogenesis and a compromised ability to withstand force. This deficiency leads to an upregulation of adipogenic marker genes PPARγ and CCAAT/enhancer binding protein α (Cebpα) in TSPCs, along with an increase in cellular lipid deposits, thus providing new insights into the molecular basis of adipose tissue accumulation in tendinopathy.

### 4.5 Metabolic disorders

**Fig. 5:**
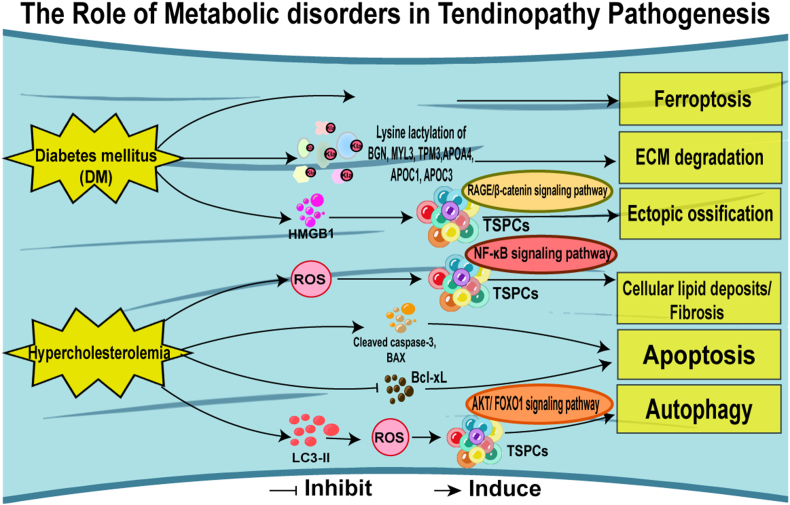
Pathogenesis of tendinopathy related to metabolic disorders. Current research indicates that diabetes mellitus can cause ferroptosis, degradation of the extracellular matrix, and ectopic ossification. Additionally, hypercholesterolemia can lead to cellular lipid deposition, fibrosis, apoptosis, and autophagy.

Increasing evidence has shown that the metabolic microenvironment is important in tendinopathy [102]. The intriguing global relevance of the connection between glucose metabolism and tendon pathology is underscored by spontaneous tendinopathy that often correlates with systemic metabolic disorders, notably diabetes mellitus [103]. Wang et al. have discovered that ferroptosis significantly contributes to tendinopathy in patients with diabetes mellitus. In vitro, experiments demonstrated that exposing TDSCs to high glucose and high-fat conditions induced a series of detrimental effects, including massive cell death, lipid peroxide accumulation, alterations in mitochondrial morphology, a reduction in mitochondrial membrane potential, iron overload, and the activation of genes associated with ferroptosis. In vivo, studies using db/db mice, a model for diabetes, revealed severe tendon injuries and elevated expressions of acyl-CoA synthetase long-chain family member 4 (ACSL4) and transferrin receptor 1 (TfR1). Significantly, these adverse effects were mitigated by treatment with ferrostatin-1, a ferroptosis inhibitor [104]. While another group of researchers did not identify specific glucose changes in the tendons, they did observe post-injury disruptions in creatinine, D-chiro-inositol, and lipid levels, suggesting a broader metabolic impact [105]. High glucose levels have also been shown to disrupt tendon homeostasis through downregulating the 5′ adenosine monophosphate-activated protein kinase/early growth response factor 1 (AMPK/EGR1) pathway and the expression of downstream tendon-related genes in tenocytes [106]. Lysine lactylation (Kla), a recently discovered post-translational modification linked to glycolysis, is elevated in both matrix proteins such as biglycan (BGN), myosin light chain 3 (MYL3), tropomyosin 3 (TPM3), and cholesterol metabolism-related proteins, including apolipoprotein A4 (APOA4), apolipoprotein C1 (APOC1), apolipoprotein C3 (APOC3) within pathological tendon sites. Despite increased Kla levels, the relative abundance of these proteins was found to be lower in pathological sites compared to normal tissues, expression of the forementioned treating cells with lactate have been shown to enhance the intracellular proteasomal protein catabolic process and downregulate the expression of the aforementioned proteins without affecting the transcription of the corresponding genes. While our understanding of the effects of lactylation on non-histone proteins is still evolving, post-translational modifications like glycation are known to induce protein misfolding and extracellular matrix dysregulation [107]. This has led to the hypothesis that lactylation may similarly impair matrix organization and cholesterol metabolism in tendinopathic tissues [108]. Additionally, under high glucose conditions, the alarmin protein HMGB1 is upregulated, activating the receptor of advanced glycation endproducts/β-catenin (RAGE/β-catenin) signaling pathway, which influences cell signaling and promotes osteogenic differentiation in TSPCs while inhibiting their tenogenic differentiation. This shift is thought to contribute to developing tendinopathy in diabetic patients [109].(see Fig. 5)

Hypercholesterolemia has also been associated with an increased risk of tendon pain and tendinopathy. Elevated cholesterol levels may suppress the expression of key tendon-related genes, including Col I, Scx, and Tnmd, in TDSCs, potentially mediated by ROS-activated NF-κB signaling [110]. Cholesterol has been shown to induce both apoptosis and autophagy in TDSCs, with the induction of apoptosis linked to increased expression of cleaved caspase-3 and Bcl2 Associated X Protein (BAX), and a decrease in B-cell lymphoma-extra large protein (Bcl-xL). The activation of autophagy, indicated by the accumulation of microtubule-associated protein 1 light chain 3-II (LC3-II), triggers ROS generation and the AKT (RAC-alpha serine/threonine-protein kinase)/FOXO1 signaling pathway. Notably, treatment with the ROS scavenger N-acetylcysteine (NAC) effectively inhibits cholesterol-induced activation of this pathway [111], offering a novel explanation for the observed link between high cholesterol levels and the heightened risk of tendinopathy in individuals with hypercholesterolemia.

## 5 Prognostic molecules of tendinopathy

Emerging research has focused on identifying molecular markers that can help predict the progression and prognosis of tendinopathy. Among which, the most widely investigated are matrix remodeling markers (in particular MMP and TIMPs families), as highly associated with pain degree, pathological progress and matrix degradation in both in human subjects and animal experiments [[112], [113], [114], [115]]. In a case–control study, the upregulation of MMP-1 and MMP-9 was highly correlated with failed healing of the rotator cuff after arthroscopic repair [116]. In genetic level, association of matrix metalloproteinases-1/3 rs1799750/rs3025058 polymorphism with increased risk of rotator cuff tear (RCT) has been reported in Brazilian and China population [117,118]. Recent studies illustrated in high-performance athletes carrying the MMP3-rs591058-T allele and other functional variants of the MMP3 gene were linked to increased risk of more episodes of tendinopathy manifestation [119,120]. Breakdown products of matrix proteins such as tenascin-C (TNC) have also been reported to accumulate in degenerate tendon. Results have been provided that recurrent defects after rotator cuff repairs are clinically relevant, and a heritable component of the disorder is plausible on the basis of a genetic association with TNC variants [119,121,122]. These results collectively suggested that variations in tendon-related matrix genes may contribute to a more susceptible ECM failure phenotype, and their interaction with environmental furtherly influence the individual risk for tendinopathy prognosis.

Given the up-to-date implications, however, few healing-related molecular indicators have been identified in perspective of the big picture. Future work should seek to identify intrinsic biomarkers that predict healing after tendinopathy, to inform the current practice and possibly identify modifiable predictors of outcome, as well as provide a scientific premise for future research into modifiable variables or interventions in the treatment of tendinopathy.

## 6 Future research direction and new treatments

The current understanding of tendinopathy's molecular mechanisms has opened avenues for developing novel diagnostic and therapeutic approaches. Magnetic resonance imaging (MRI) and ultrasound are routinely used for diagnosing tendinopathy with excellent sensitivities and specificities, yet none offers perfect reliability or the ability to discern tissue type or severity [123]. As promising alternatives, latest strategy utilized combination of histological methods and single cell sequencing to identify the progenitor population and the pathway contributed to neonatal-to-adult and injury-to-regeneration switching that regulates tendon maturation [[124], [125], [126]]. Therefore, there should be a focus on developing histological and molecular indicators for early diagnosis and for monitoring both the tissue scale and cell level transformation throughout the tendinopathy pathological stages.

Secondly, given the multifactorial nature of tendinopathy, treatments should aim for a multitargeted strategy that addresses the complex interplay of mechanical, inflammatory, oxidative, and metabolic factors. Efforts to conduct large-scale randomized controlled trials to confirm the efficacy of emerging treatments are equally crucial for overcoming current challenges and unlocking the full therapeutic potential of these advanced treatments.

NSAIDs, exemplified by aspirin, have been shown to modulate the JNK/STAT-3 signaling pathway, thereby inhibiting inflammation and scar formation, and reducing the risk of re-rupture in injured tendons [127]. NSAIDs like Celecoxib have demonstrated the potential to protect young TDSCs from senescence induced by age-associated inflammation, or "inflame-aging," through the inhibition of the NF-κB pathway, thus preserving their tenogenic differentiation potential [128]. The use of small interfering RNA (siRNA)-loaded gold nanoclusters (AuNC-siRNA) to target the inhibition of the I kappa B kinase β (IKKβ)/NF-κB pathway represents a promising new direction for treating degenerative rotator cuff tendinopathy [129].

Furthermore, synthetic glucocorticoids, potent steroidal anti-inflammatory agents, are widely utilized in managing a range of painful and inflammatory conditions across various tissues. However, the use of these drugs must be approached with prudence due to their potential to induce cellular senescence. This senescence, triggered by the inhibition of sirtuin 1 and the activation of the Tumor Protein 53/Tumor Protein 21 (p53/p21) pathway, may result in long-term degenerative alterations within the tendon tissue, thereby compromising its structural integrity and function over time [130]. Consequently, there is a need for careful consideration when prescribing glucocorticoids, especially in scenarios where the risk of senescence induction could surpass the immediate anti-inflammatory benefits.

By demystifying the molecular underpinnings of various treatment modalities, we can enhance our understanding of effective interventions for chronic tendinopathic lesions. Moreover, this knowledge equips us to navigate treatment strategies that minimize the risk of exacerbating the existing tissue pathology. It is this balance between therapeutic efficacy and safety that will guide the development of more judicious and personalized treatment approaches for tendinopathy.

The development of biomaterials that mimic the tendon's ECM could provide a conducive environment for tendon repair, especially when combined with stem cell therapies. Furthermore, understanding the niche of TSPCs and their role in tendon healing could lead to cell-based therapies. An innovative study presents a breakthrough delivery system for TSPCs encapsulated within a DNA hydrogel that serves as an artificial ECM. This TSPCs-Gel system supports a favorable microenvironment for cell proliferation and shields cells from mechanical forces such as shear. The encapsulation technique effectively localizes TSPCs to the tendon, significantly enhancing their retention time. Moreover, by employing RNA sequencing, the study uncovers the TSPCs-Gel's underlying healing mechanisms, identifying potential gene and signaling pathway targets that are pivotal for crafting future clinical treatment strategies [131]. The harnessing of stem cell potential for regenerating damaged tendon tissue is a frontier in regenerative medicine. In a tendinopathy mouse model, adipose-derived mesenchymal stem cells and extracellular vesicles from younger mice (ADMSC young-EVs) have demonstrated the capacity to ameliorate affected tendons' pathological structural, functional, and biomechanical properties. This suggests that the rejuvenating effects of ADMSC young-EVs could be a therapeutic boon for tendinopathy [132]. Furthermore, the transfer of mitochondria from mesenchymal stem cells (MSCs) to tenocytes has been shown to reduce apoptosis, promote cell proliferation, and restore mitochondrial function in tenocytes compromised by hydrogen peroxide (H_2_O_2_) [133]. Induced pluripotent stem cell-derived mesenchymal stem cells (iMSC-sEVs) have also shown promise in alleviating tendinopathy-related pain by inhibiting mast cell activation through the Hypoxia-inducible factor 1 (HIF-1) signaling pathway, indicating their potential in managing tendinopathy and other pain-related conditions [134]. Human hair follicle-derived mesenchymal stem cells (hHF-MSCs) are emerging as a valuable therapeutic asset for tendon repair, with their ability to modulate the tendon's ECM environment, which is possibly achieved through the upregulation of tenascin-C and downregulation of MMP-9 [135].

Identifying key miRNAs and lncRNAs involved in tendinopathy opens new horizons for gene therapy, offering a means to modulate the expression of these molecules and foster healing. A recent study shows that microRNA-146a (miR-146a) expression is inversely correlated with tendinopathy progression, with lower levels of miR-146a presence as the condition worsens. Overexpression of miR-146a has been found to protect tendinopathic tenocytes from SASPs and senescence through the Interleukin-1 receptor-associated kinase 4 (IRAK-4)/TNF receptor associated factor 6 (TRAF6)/NF-kB pathway [136]. The principle has been demonstrated that locally delivered miR29a therapy can enhance early tendon healing, presenting a novel therapeutic approach [137]. Additionally, miR-148a-3p has been identified as a significant player in tendinopathy development, with its increased levels in patients and its role in suppressing Kruppel-like factor 6 (KLF6) and upregulating thrombospondin-4 (Tsp-4), thereby influencing angiogenesis [138]. The discovery of the AC108925/miR-146a-3p axis as a regulator of the osteogenic differentiation of human TDSCs adds another layer to the gene therapy approach for tendinopathy [139].

Further research into how mechanical loading influences tendon cell behavior and ECM organization is also essential for understanding the onset and progression of tendinopathy. Recent research has uncovered that the overexpression of Yes-associated protein (Yap) plays a dual role in cellular response to mechanical stress. It not only shields against the activation of a comprehensive catabolic program that would typically follow the loss of cellular tension, but it also maintains the integrity of the chromatin structure, preventing it from being altered by mechanical forces. These findings elucidate the intricate mechanisms through which mechanoepigenetic signals, via the Yap/Tafazzin protein (Taz) axis, govern the function of tendon cells, offering new insights into the interplay between mechanical cues and cellular regulation [140]. Current studies have demonstrated the protective effects of dehydroepiandrosterone against oxidative stress in tenocytes under high glucose conditions, the potential of antioxidant and anti-inflammatory hydrogel microspheres for the in-situ treatment of tendinopathy, and the importance of reactive oxygen species scavenging in modulating the differentiation of tendon stem and progenitor cells to prevent ectopic calcification in tendinopathies [[141], [142], [143]].

Elucidating the role of oxidative stress in tendinopathy and identifying more novel antioxidants could lead to the application of therapeutic agents that protect tendon cells. Given the link between metabolic disorders and tendinopathy, research into the metabolic reprogramming of tendon cells and its impact on tendon homeostasis is crucial. Seven key metabolites were identified as biomarkers of supraspinatus tendon progression in diabetes. The purine metabolism pathway has been pinpointed as significantly impacted by diabetes in this context [144]. Physical therapy and rehabilitation exercises play a pivotal role in managing tendinopathies. These non-surgical interventions aim to alleviate pain, reduce inflammation, improve blood supply in the affected area, and enhance the overall function of the tendon. The introduction of percutaneous needle electrolysis, applying a galvanic current to activate the nucleotide-binding oligomerization domain, leucine-rich repeat and pyrin domain-containing 3 (NLRP3) inflammasome and promote collagen-mediated tendon regeneration, represents an innovative non-surgical approach [145].(see Table 1)

**Table 1:** Molecular pathogenesis of tendinopathy.

Factor	Effects on tendon	Molecular signaling pathway
Mechanical Loading		
Overload treadmill	Decreased cross‐sectional area of the supraspinatus tendon, increased glycosaminoglycan	A significant upregulation of PTGS2 [72]
9 % strain overload	ND^a^	The extracellular mitochondrial particles induced macrophage chemotaxis and increased the production of proinflammatory cytokines, including IL-6, CXCL1, and IL-18 [75]
Cyclic mechanical stretch	Ectopic ossification in tendon at the late stages of tendinopathy	Interleukin-1β acts as an upstream regulator of miR-337-3p connecting mechanical stress to the miRNA's expression and the target genes of miR-337-3p, NADPH oxidase 4, and insulin receptor substrate 1, activated chondro-osteogenic differentiation of TDSCs through JNK and ERK signaling, respectively [76]
Mechanical underloading	Increased osteogenic gene expression and heterotopic ossification alongside diminished biomechanical strength	This phenomenon is associated with increased β-catenin accumulation and altered phosphorylation patterns [77]
Inflammation		
Diseased Achilles-tendon-derived stromal cells	A complex tissue inflammation signature and increased vascularity	Expressing target molecules from interferon, NF-κB, STAT-6 and GCR activation pathways [60]
IL -1β	Diseased tendon-derived stromal cells display dysregulated resolution responses	Increased 15-prostaglandin dehydrogenase expression as well as increased concentrations of both 15-epi-LXA4 and MaR1 further metabolites, 15-oxo-LXA4and 14-oxo-MaR1 [80]
HMGB-1	Inflammatory and matrix changes	High-mobility group protein B1 appears to be intricately involved in tendon cell inflammation and matrix regulation through TLR4 signaling [82]
S100A8 and S100A9	Inflammatory signature	Their participation in a positive feedback loop that amplifies leukocyte recruitment and the secretion of pro-inflammatory cytokines [84]
Heightened prostacyclin receptor expression and enzymes involved in prostaglandin biosynthesis	Inflammation and pain	PGIS was found to co-localize with cells expressing PDPN and the nociceptive neuromodulator NMDAR-1, indicating that PGE2 may perpetuate inflammation and pain [85]
Subacromial impingement	Increased cellularity and disorganized collagen fibers	The elevated levels of Spp1 could indicate an upregulation and differentiation of macrophages in the setting of rotator cuff tendinopathy [74]
Oxidative stress		
Excess ROS	Cellular dysfunction and tissue damage	PRDX5 may play a protective role against oxidative stress during this pathophysiological process [88]
S100A11, PLIN4, HYOU1 and CLIC1	Pain, dysfunction, and chronic inflammation	ND^a^ [89]
Pparg and Sox9	The expression of Col III and the Col III/Col I ratio was elevated	Aberrant differentiation in tendon [90]
A specific cell subpopulation with low PRDX2 expression	The inhibition of the proliferation TDSCs	The FOXO1-PRDX2-TNF axis [91]
The loss of Sod1	The downregulation of collagen and a decrease in the mechanical properties	ND^a^ [92]
Mitochondrial dysfunction	Decrease in failure force of the supraspinatus tendons	Mitochondrial Sod is diminished [93]
MMP1	Tendon matrix degradation	MMP1 activity is upregulated by phosphorylated JNK induced by oxidative stress [94]
HSPs	Leading to the cascade of stress-activated programmed cell death and degeneration	The reduction of the apoptotic gene cFLIP and the increase in caspases 3 and 8 and HSPs 27 and 70 [96]
Tendon Cell Senescence		
Aging	The loss of ECM integrity	Both pro- and active forms of MMP-2 and MMP-9 were elevated, and the mRNA levels of tissue inhibitors of TIMP-1 and TIMP-2 were decreased in aged tissues compared to their younger counterparts [97,98]
PGE2, PGF2α, LXA4	Inflammatory signature	Aging individuals have a diminished capacity to resolve tendon inflammation via the FPR2/ALX receptor [99]
P16	The capacity for tenogenic differentiation in TSPCs declines with age	The p16/miR-217/EGR1 pathway modulates age-related tenogenic differentiation in tendon stem/progenitor cells [100]
Sparc	Adipocyte accumulation	An upregulation of adipogenic marker genes PPARγ and Cebpα in TSPCs [101]
Metabolic disorders		
Ferroptosis	Massive cell death, lipid peroxide accumulation, alterations in mitochondrial morphology	Elevated expressions of ACSL4and TfR1 [104]
High glucose	Disrupt tendon homeostasis	Downregulating the AMPK/EGR1 pathway and the expression of downstream tendon-related genes in tenocyte [106]
BGN, MYL3, TPM3, and cholesterol metabolism-related proteins	Extracellular matrix dysregulation	Kla is elevated in these protein [107]
HMGB	Leading to osteogenic differentiation	Under high glucose conditions, HMGB1 activates the RAGE/β-catenin signaling pathway [109]
Elevated cholesterol levels	key tendon-related genes were suppressed	Potentially mediated by ROS-activated NF-κB signaling [110]
Elevated cholesterol levels	The activation of autophagy	The accumulation of LC3-II triggers ROS generation and the AKT/FOXO1 signaling pathway [111]

a ND, not determined.

## 7 Conclusions

Tendinopathy is a complex and multifactorial disease that affects tendon function and structure. The molecular mechanisms underlying its pathogenesis involve intricate interactions between mechanical stress, inflammation, oxidative stress, metabolic disorders, and so on. The heterogeneity of tendinopathy necessitates a personalized approach to treatment, considering individual patient factors such as age, activity level, and comorbidities. Tendinopathy substantially impacts patients' quality of life. Although current clinical treatments provide symptomatic relief, they have shown limited success in fully restoring tendon function. The efficacy of these treatments is still inconsistent, presenting a challenge in identifying universally effective treatments. The advent of molecular and cellular therapies holds promise for more effective interventions. A deeper understanding of the molecular pathways involved in tendinopathy will inform the development of targeted treatments and improve our ability to predict and prevent its onset. However, the mechanisms involved in tendon pathologies remain largely unknown. The future of tendinopathy research lies in integrating omics data, advanced imaging techniques, and biomechanical studies to create a comprehensive view of the disease process. New and innovative treatments like gene therapy and epigenetic modulation are paving the way for targeted interventions in tendon degeneration and repair by focusing on specific molecular pathways [129,[136], [137], [138]]. By leveraging this knowledge, the scientific community can work towards innovative therapies coupled with rigorous clinical validation that address the root causes of tendinopathy, ultimately improving patient outcomes and reducing the socioeconomic burden of this prevalent musculoskeletal disorder.

## 8 Limitations

In our comprehensive review of the literature, we identified several limitations that are pertinent to the current understanding of tendinopathy. Firstly, the heterogeneity in study designs and methodologies across the studies we reviewed makes it challenging to draw definitive conclusions. This variability includes differences in patient populations, animal models, sample sizes, and diagnostic criteria for tendinopathy, which can lead to inconsistent findings. Secondly, we acknowledge the inherent limitations due to the decision not to include a discussion on prognostic factors of tendinopathy. This decision was made in response to the inconsistencies and conflicts present in the literature, which we believe necessitate a cautious approach to interpreting preliminary and conflicting data. This underscores the urgent need for further research, particularly well-designed studies with robust methodologies, to advance our understanding of the prognostic factors associated with tendinopathy and to guide clinical practice.

## Data availability

No datasets were generated or analyzed during the current study.

## Ethics approval and consent to participate

Not applicable.

## Consent for publication

Not applicable.

## Author contributions

Draft and figure preparation, Z.X.; Bibliographic retrieval, W.H.; Draft organization, submission and revision, T.Z.; Review and editing, R.C., T.S.; Funding acquisition, R.C., T.S. All authors have read and agreed to the published version of the manuscript.

## Statement

Neither the entire study nor any part of it has been published, accepted for publication, or under consideration for publication elsewhere.

## Funding

Z.H. is supported by the 10.13039/501100004543China Scholarship Council (CSC). T.Z. is funded by Sino-German (CSC-DAAD) Postdoc Scholarship Program. R.C. is funded by the Taishan Scholarship (NO. tsqn202306399) Project. This review was financed by the University Clinic Heidelberg and the European Wellness Academy (ASIA PACIFIC) and Taishan Scholarship (NO. tsqn202306399) Project.

## Declaration of competing interest

The authors have no conflicts of interest to disclose in relation to this article.
